# Morphological, Morphometric, and Histological Evaluation of the Placenta in Cases of Intrauterine Fetal Death

**DOI:** 10.7759/cureus.62871

**Published:** 2024-06-21

**Authors:** Mah Paiker, Kamil Khan, Dewanshi Mishra, Stuti Tandon, Abeer Khan, Asma Nigar, Syed Fiza Mustaqueem, Mahboobul Haque

**Affiliations:** 1 Anatomy, Integral Institute of Medical Sciences and Research, Integral University, Lucknow, IND; 2 Obstetrics, Integral Institute of Medical Sciences and Research, Integral University, Lucknow, IND; 3 Pathology, Integral Institute of Medical Sciences and Research, Integral University, Lucknow, IND; 4 Anatomy, All India Institute of Medical Sciences, Patna, Patna, IND

**Keywords:** intrauterine fetal death, placenta, histology, morphology, morphometry

## Abstract

Background

The human placenta is a remarkable organ that develops during pregnancy and is crucial in supporting fetal growth and development. The placenta supplies oxygen and nutrients to the fetus and removes waste products from the fetal bloodstream. It also produces hormones that support pregnancy, such as human chorionic gonadotropin, progesterone, and estrogen. Placental insufficiency occurs when the placenta cannot deliver adequate nutrients and oxygen to the fetus. This can result in intrauterine fetal death (IUFD), stillbirth, intrauterine growth restriction, low birth weight, and premature birth. It can also be associated with developmental delays or long-term health issues for the baby. This study aimed to assess the morphologic, morphometric, and histologic changes in the placenta associated with IUFD and compare it with the placenta of live births.

Methodology

This study was conducted at the Department of Anatomy in collaboration with the Department of Obstetrics and Gynaecology, Integral Institute of Medical Sciences and Research and King George’s Medical University Lucknow, where 60 placentas were studied. Placentas were further categorized into the following two groups: Group A, the study group in which placentas from IUFD were taken (n = 30), and Group B, the control group where placentas from live births were taken (n = 30). Morphological and morphometric features of both groups were recorded and compared. Histological features of placentas from IUFD (Group A) were examined after hematoxylin and eosin staining.

Results

A total of 60 placentas were observed (Group A and Group B). In Group A (IUFD) and Group B (control group), most pregnancies were multigravidas. Round-shaped placentas were the most common type in both groups (Group A = 46.67%, Group B = 66.67%). The average thickness of placentas from Group A (IUFD) cases was significantly reduced (mean thickness = 1.17 ± 0.07 cm) compared to controls in Group B (mean thickness = 2.04 ± 0.93 cm). The p-value obtained was significant at 0.0001. There was a notable reduction in the average placental diameter in Group A (mean diameter = 241.73 ± 65.54 cm) compared to Group B (mean diameter = 263.72 ± 162.67 cm). The p-value obtained was not significant at 0.49. On histopathological examination of the placentas of Group A (IUFD), perivillous fibrin deposition and high-grade calcification were seen in a significantly high number of placentas (70% and 60%, respectively).

Conclusions

The knowledge of the placenta’s morphologic, morphometric, and histologic changes can be utilized to establish the cause of fetal death. In instances of fetal growth limitation and fetal demise that are clinically inexplicable, they can also explain the causes.

## Introduction

The placenta is an intricate and vital organ during pregnancy, coordinating a wide range of functions throughout this period. Alongside the fetus, the placenta undergoes significant transformation and growth from its early development to the end of pregnancy [1]. Although the placenta is present only during gestation, its impact extends beyond this period. The fetus perceives the placenta as a reflection of the external environment, and the signals it receives through this organ can have significant consequences for the newborn and even into adulthood [2].

In a study of 1,064 intrauterine deaths, the placenta was submitted for examination in 946 (89%) cases as part of the autopsy. Among these, 307 (32%) cases were attributed to abnormalities of the placenta, cord, or membranes [3].

Between weeks 10 and 12 post-fertilization, the placenta typically weighs around 51 g. By the time of delivery, the mature placenta is generally a discoid organ weighing between 500 and 600 g, with a diameter of 22 cm and a thickness of 2 to 4 cm. These measurements can vary in cases of abnormal or pathological pregnancies [4,5].

Intrauterine fetal death (IUFD) is a profoundly distressing event with significant emotional, psychological, and social implications for affected families and healthcare providers. Despite advances in maternal and fetal medicine, the incidence of IUFD has not significantly declined. Management of pregnancies with an intrauterine death in situ requires careful assessment of maternal and fetal health, as well as consideration of the potential risks associated with the presence of the device. Close monitoring throughout pregnancy, including regular ultrasound examinations to evaluate placental function and fetal growth, is essential. Research on placental insufficiency, specifically in IUFD pregnancies, is limited, with studies providing insights into potential mechanisms, clinical observations, and management considerations.

This study was conducted to assess the morphologic, morphometric, and histologic changes in the placenta associated with IUFD and compare them with the placenta of live births.

## Materials and methods

This observational study was conducted in the Department of Anatomy in collaboration with the Department of Obstetrics and Gynaecology, Integral Institute of Medical Sciences and Research (IIMSR), Lucknow, and the Department of Obstetrics and Gynaecology, King George’s Medical University (KGMU), Lucknow. The study was conducted after obtaining ethical clearance from the Institutional Ethics Committees of KGMU and IIMSR vide reference codes 122nd ECMIIB-Ph.D/PI and IECLIIMS&RL2022, respectively.

Methodology

A total of 60 placentas of normal or cesarean deliveries were obtained from the Department of Obstetrics and Gynaecology of IIMSR and KGMU over one year (April 2023 to March 2024) after obtaining informed consent from the patient. The placentas were divided into the following two groups: 30 placentas were of the patients with IUFD, the study group (Group A), and 30 placentas were from live births, the control group (Group B). A brief antenatal history was obtained from the patients. The placenta of patients with high-risk pregnancies, spontaneous abortion, placenta without an umbilical cord, placentas of patients who underwent medical termination, and those with gestational age <28 weeks were excluded from the study. The procured specimens were brought to the Department of Anatomy, IIMSR. The placenta was rinsed under flowing tap water, and an absorbent towel was used to remove blood clots.

A gross examination of the placentas was done. The weight of the placentas of both Groups A and B was measured in grams using an electronic balance. The placental morphology was observed and the presence of any accessory lobes was recorded. The maximum diameter of the placentas was measured using a metallic scale, following which a measurement perpendicular to the maximum diameter was taken. The average of the two measurements was calculated and the placental diameter was determined.

**Figure 1:**
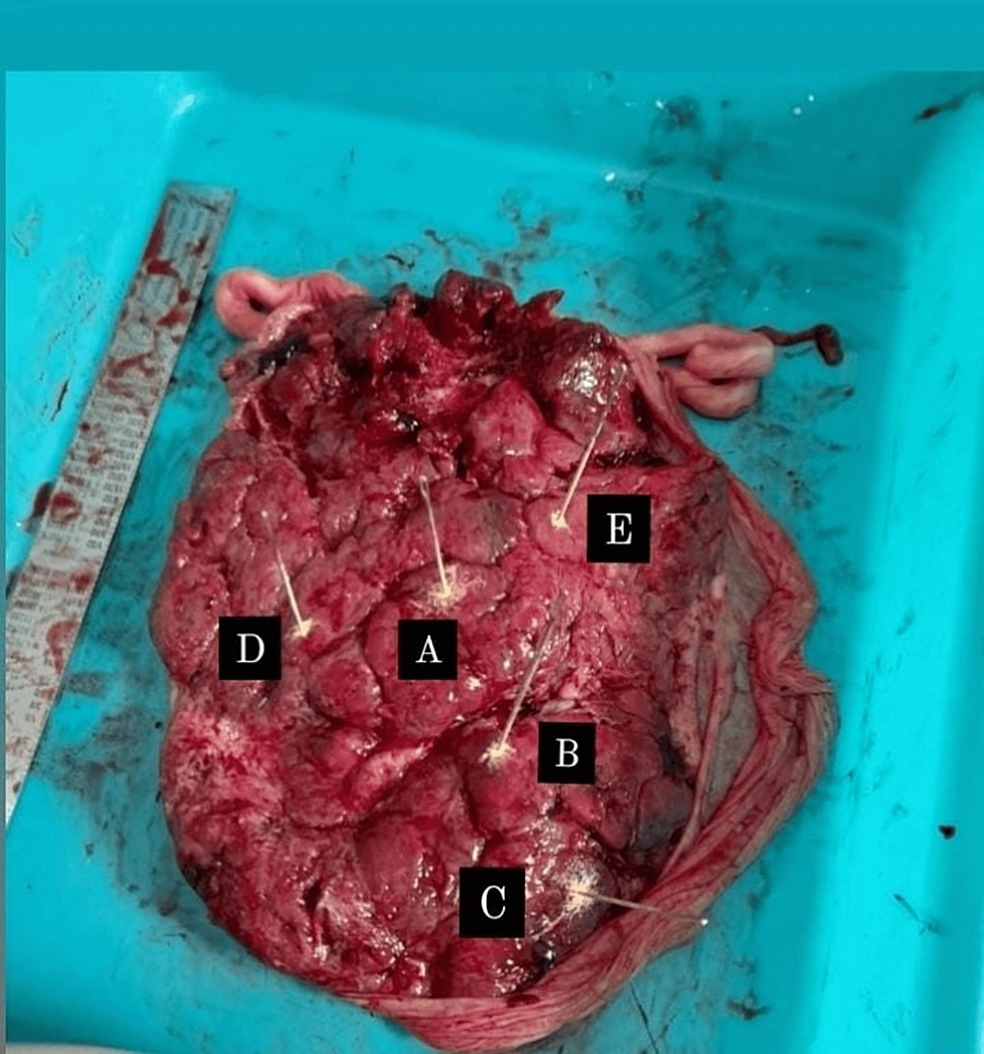
Measurement of placental thickness. The measurements are taken through the points as follows: (A) center, (B) intermediate, (C) peripheral, and (D & E) random.

To evaluate the thickness a long needle was used to measure the placental thickness at five regions. The five points included the center, the most peripheral part, the intermediate area between the two, and two random areas. The mean of the five measurements was used to calculate the placental thickness (in cm) (Figure 1).

The site of the umbilical cord attachment was observed and recorded in both Groups A and B. The attachment sites were classified as central, velamentous, eccentric, and battledore. The length and diameter of the umbilical cord were also recorded using a metal scale and divider, respectively.

The placentae from Group A were stored in a jar with 10% formalin for fixation. After 48 hours, a 2 × 2 cm thick section was cut from the placenta using a scalpel for the histological study at three different locations. One section was obtained from the insertion site of the umbilical cord (central), one section from the peripheral part, and one section from the intermediate part between the two sites mentioned above. The sections were then subjected to histological processing.

The hematoxylin and eosin-stained sections of Group A placentas were examined for placental calcification, perivillous fibrin deposition, villous shape and size, syncytial knots, acute intervillitis and perivillitis, villous vascularity, placental ischemia and infarction, placental villous immaturity, increased terminal villi, and dilated vascular channels with granulation.

Other placental abnormalities that were observed and recorded included retro-placental lobes, accessory-placental lobes, placental cysts on the membranes, retro-placental hematoma, edema, acute funisitis, and placenta succenturia.

Statistical analysis

The data obtained were tabulated in a Microsoft Excel sheet (Microsoft Corp., Redmond, WA, USA). The statistical analysis was done using ISPSS Statistics for Windows, version 23.0 (IBM Corp, Armonk, NY, USA).

## Results

Patient profile

In Group A, out of 30 cases, 20 (66.6%) pregnancies were multigravids and 10 (33.3%) were primigravids. In Group B, out of 30 cases, 17 (56.67%) pregnancies were multigravids. The mean maternal age was 28.03 ± 4.62 years in Group A and 25.96 ± 3.58 in Group B.

Placental morphology and morphometry

The placentas from IUFD cases were generally smaller and lighter compared to the control group. The average weight of placentas in Group A was lower (mean weight = 325 ± 40.87 g) compared to Group B (mean weight = 344 ± 67.1), and the p-value obtained was not significant (0.35). There was a notable reduction in the average placental diameter in Group A (mean diameter = 241.73 ± 65.54 cm) compared to Group B (mean diameter = 263.72 ± 162.67 cm), and the p-value obtained was not significant (0.49). The average thickness of placentas in Group A cases was significantly reduced (mean thickness = 1.17 ± 0.07 cm) compared to Group B (mean thickness = 2.04 ± 0.93 cm), and the p-value obtained was significant (0.0001) (Table 1).

**Table 1:** Morphometric examination of the placenta in Group A and Group B.

Morphometry of the placenta	Group A (n = 30)	Group B (n = 30)	P-value
Placental weight (g)	325.3 ± 40.87	344.5 ± 67.11	0.35
Placental diameter (cm)	241.73 ± 65.54	263.72 ± 162.67	0.49
Placental thickness (cm)	1.17 ± 0.07	2.04 ± 0.93	0.0001

Out of 30 placentas of IUFD (Group A), 14 (46.67%) were round, and 10 (33.33%) were irregular in shape. In Group B, out of 30 placentas, 20 (66.67%) were round, followed by six (20%) oval-shaped, and four (13.33%) heart-shaped placentas (Table 2).

**Table 2:** Placental shapes in Group A and Group B.

Placental shapes	Group A (n = 30)	Group B (n = 30)
Round	14 (46.67%)	20 (66.675)
Oval	5 (16.67%)	6 (20%)
Heart	1 (3.33%)	4 (13.33%)
Irregular	10 (33.33%)	0

Umbilical cord morphology and morphometry

The mean umbilical cord length recorded in Group A and Group B was 20.79 ± 2.91 and 28.82 ± 10.8 cm, respectively. The p-value was significant (0.0002). The umbilical cord diameter in Group A was 1.03 ± 0.15 cms whereas in Group B was 1.13 ± 0.24. The p-value obtained was not statistically significant (0.05) (Table 3).

**Table 3:** Morphometric examination of the umbilical cord in Group A and Group B.

Morphometry of the umbilical cord	Group A (n = 30)	Group B (n = 30)	P-value
Length (cm)	20.79 ± 2.91	28.82 ± 10.8	0.0002
Diameter (cm)	1.03 ± 0.15	1.13 ± 0.24	0.05

**Figure 2:**
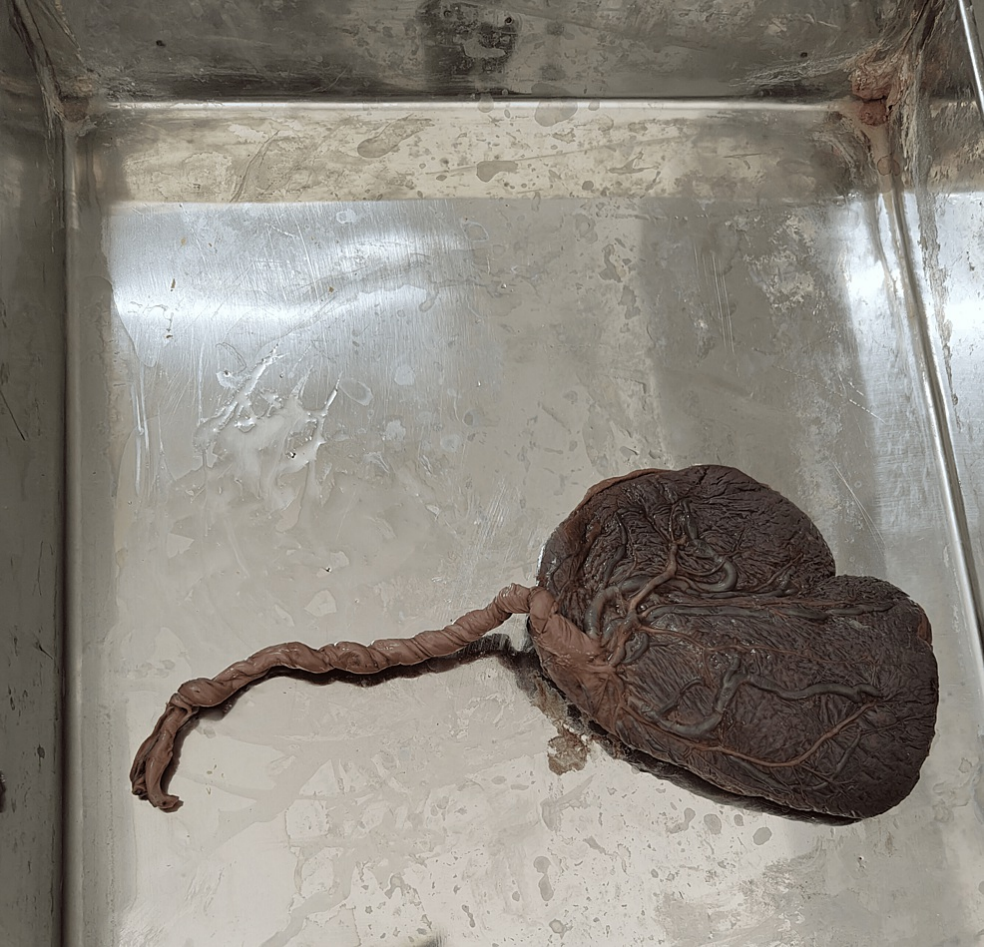
Velamentous type of umbilical cord insertion.

In the present study, 36.67% of umbilical cords showed a velamentous type of insertion in Group A, whereas the central type of insertion was found to be more common in Group B (Table 4, Figure 2).

**Table 4:** Umbilical cord insertion in Group A and Group B.

Umbilical cord insertion	Group A (n = 30)	Group B (n = 30)
Central	1 (3.33%)	11 (36.67%)
Eccentric	9 (30%)	4 (13.33%)
Marginal	7 (23.33%)	6 (20%)
Battledore	2 (6.67%)	7 (23.33%)
Velamentous	11 (36.67%)	2 (6.67%)

Histological findings of the placenta

**Figure 3:**
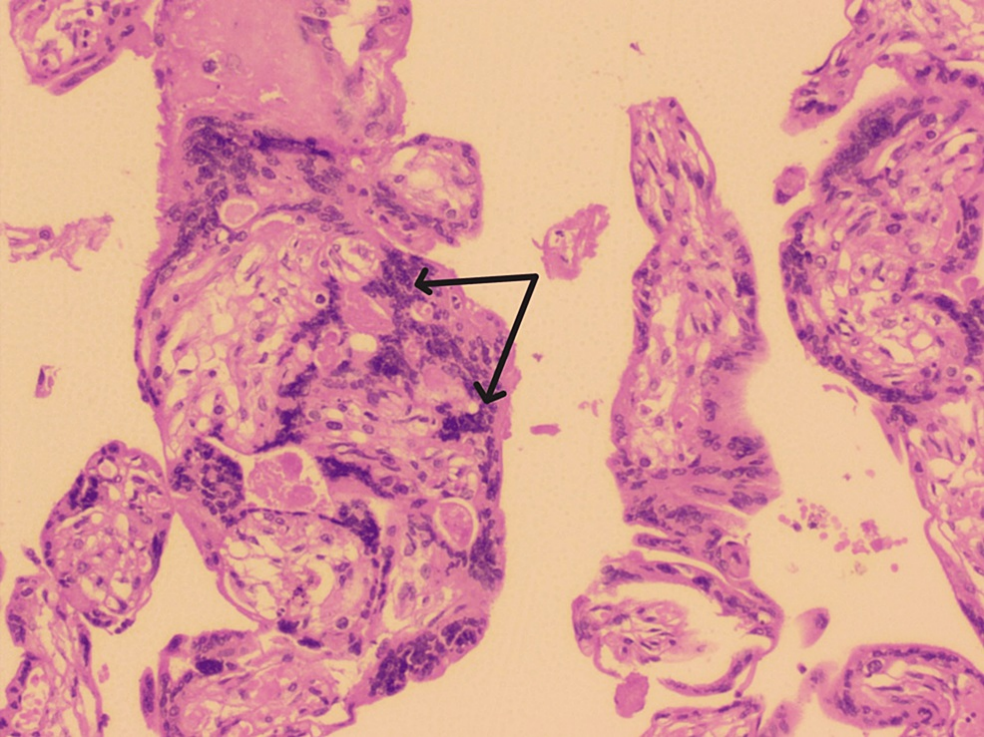
Histological image of the placenta showing perivillous fibrin deposition (black arrows).

On histological examination, a significantly high number of perivillous fibrin deposition (70%) was found in Group A placentas (Figure 3).

**Figure 4:**
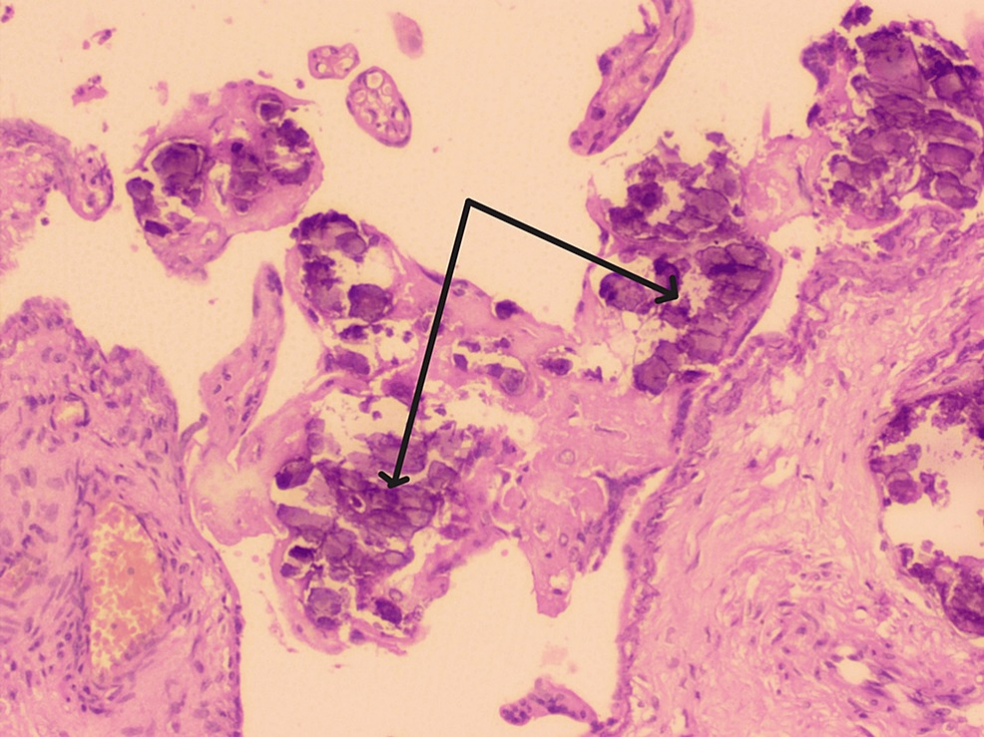
Histological image of the placenta showing calcification (black arrows).

High-grade calcification was seen in 60% of the placentas in Group A (Figure 4).

**Figure 5:**
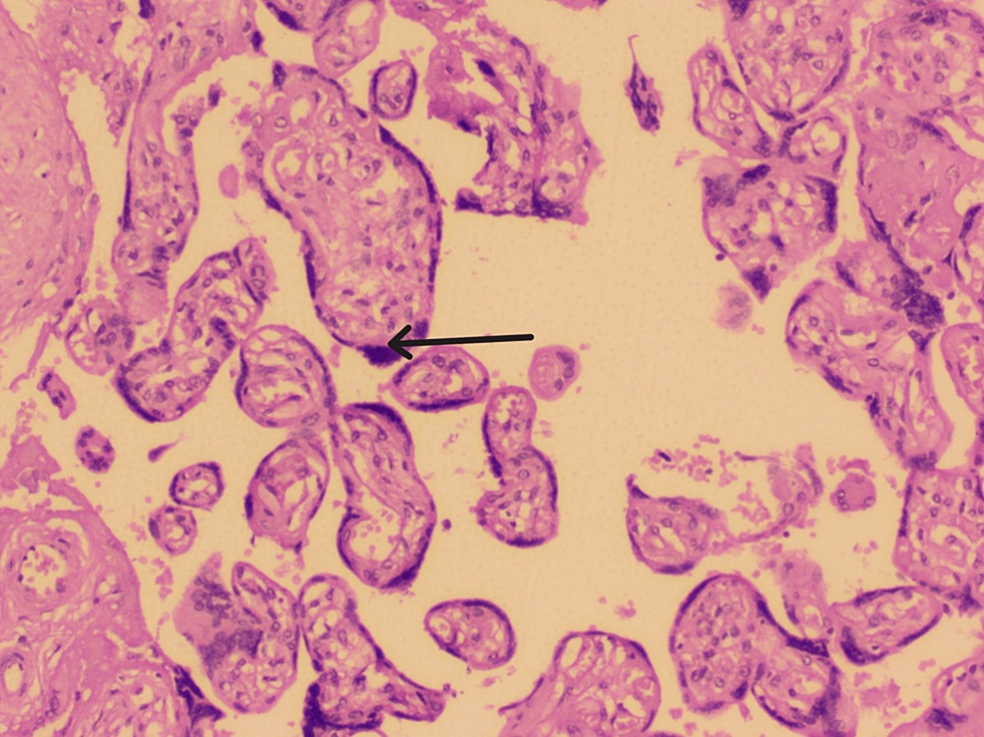
Histological image of the placenta showing syncytial knots (black arrow).

We also observed a marked variation in villous shape and size in 36.67% of placentas. Overall, 40% of placentas showed increased syncytial knots, and an increase in villous vascularity was seen in 30% of placentas (Figure 5).

Villous/Parenchymal infarction was seen in 20% of IUFD pregnancies in the study. Other findings such as decreased syncytial knots, acute intervillitis and perivillitis, placental ischemia, placental villous immaturity, increased terminal villi, and dilated vascular channels with large foci of granulation were seen in the present study (Table 5).

**Table 5:** Histological findings of the placentas in Group A.

Histological findings of Group A placentas (n = 30)	n (%)
Increased syncytial knots	12 (40%)
Decreased syncytial knots	3 (10%)
Perivillous fibrin deposition	21 (70%)
Acute intervillitis and perivillitis	3 (10%)
Villous/Parenchymal infarction	6 (20%)
Intervillous sclerosis	6 (20%)
Increased villous vascularity	9 (30%)
Placental ischemia	3 (10%)
Placental villous immaturity	3 (10%)
Increased terminal villi	3 (10%)
Marked variation in villous shape and size	11 (36.67%)
Dilated vascular channels with large foci of granulation	3 (10%)
High-grade calcifications	18 (60%)

## Discussion

The examination of morphologic, morphometric, and histologic features of the placenta in IUFD cases offers significant insights into the potential causes and contributing factors of fetal demise. This detailed analysis enhances our understanding of placental pathology and its critical role in fetal health and development. The present study was conducted on 30 placentas obtained from IUFD cases and 30 placentas from live births.

In a study, 64% of patients presenting with IUFD were multigravida, while 35.8% of patients were primigravida [6]. Kalewad et al. reported that pregnancy in women aged 35 years and above is associated with higher maternal and perinatal mortality [7]. The study by de la Rochebrochard et al. suggested that among women older than 35 years of age, the risk of fetal death increases [8]. In the present study, the mean age of the mothers was 28.03 ± 4.62 years.

Baghel et al. reported that placental thickness is an important parameter in the estimation of fetal age along with other parameters. Thin placentas ≤29 mm at 32 weeks and ≤31mm at 36 weeks are associated with increased morbidity [9]. Nagpal et al. observed that neonatal outcomes were better in women with normal placental thickness than those with thick placenta [10]. In contrast, Miwa et al. and Verma et al. observed that placental thickness was greater in patients with fetal growth retardation compared to normal thickness [11,12]. In this study, the diameter and thickness of the placenta in IUFD cases were 241.73 ± 65.54 cm and 1.17 ± 0.07 cm, respectively. It was observed that the diameter of the placenta of IUFD cases was less than the diameter of the placenta of live births. The thickness of the placenta was significantly reduced in placentas of patients with IUFD.

Siargkas et al. reported that velamentous cord insertion is associated with several adverse perinatal outcomes, including stillbirth and intrauterine death [13]. Nkwabong et al. reported that marginal cord insertion is associated with increased maternal, fetal, and adverse neonatal morbidities [14]. Räisänen et al. observed that velamentous cord insertion increases along with an increase in fertility problems and maternal obesity. Velamentous cord insertion presents a moderate risk of prematurity and impaired fetal growth [15]. A study reported that placental attachment of the umbilical cord varies from central to eccentric to marginal with few velamentous attachments [16]. Paiker et al. observed a narrow diameter of the umbilical cord in hypertensive cases [17]. The present study showed a significant number of cord insertions to be velamentous in cases of IUFD. The diameter of the umbilical cord in cases of intrauterine death was less than the diameters recorded in placentas from live births.

According to Sharma et al., cases that had short and long cords constituted abnormal cord length, and these cases had a higher incidence of cord complications, increased incidence of operative interference, intrapartum complications, increased fetal heart rate abnormalities, and more chances of birth asphyxia [18]. According to Taweevisit et al., both excessively long umbilical cords and fetal thrombotic vasculopathy (FTV) are associated with poor perinatal outcomes, such as fetal mortality and long-term neurological impairments. They further added that the etiology of these illnesses has not been very clear but is most likely complicated. Fetal death and FTV have been associated with long umbilical cords [19]. In the present study, umbilical cord length recorded in IUFD cases was less compared to live births, which may be variable depending upon the site it was cut during the delivery.

Kotgirwar et al. noted that syncytial knots indicate impaired fetal circulation, while fibrinoid necrosis is considered a marker of immunological reactions affecting trophoblastic cells. [20]. According to Bane et al., perivillous fibrin deposition reduces the nutritional supply of the fetus, which results in intrauterine growth restriction [21]. Günyeli et al. reported that a barrier separating the circulation of the mother and the fetus may be created by perivillous fibrin depositions [22]. Ch et al. observed that 37% of intrauterine death placentas showed intravascular thrombosis [23]. In this study, during the histological examination, perivillous fibrin deposition was seen in 70% of placentas, high-grade calcifications were seen in 60% of placentas, and marked variation in villous shape and size was seen in 36.67% of placentas. Increased syncytial knots were seen in 40% of placentas, increased villous vascularity of placentas, and villous/parenchymal infarction was also seen in intrauterine death pregnancies. Other findings such as acute intervillitis and perivillitis, placental ischemia, placental villous immaturity, increased terminal villi, and dilated vascular channels with large foci of granulation were also observed in a few cases.

Limitations of the study

The limited number of cases can reduce the statistical power of the study, making it difficult to draw generalizable conclusions. Intrauterine death has multiple etiologies, including genetic, infectious, maternal, and environmental factors. This heterogeneity can complicate the analysis and interpretation of placental changes. The time interval between fetal death and delivery can influence the placental morphology and histology, leading to postmortem changes that may confound results.

## Conclusions

In cases of IUFD, the placenta often holds crucial insights into the potential causes. Examination of the placenta can reveal signs of placental insufficiency, infections, clotting disorders, or other pathologies that might have compromised fetal health. Understanding placental abnormalities can provide vital information for grieving parents, guiding them through their loss and informing future pregnancies. Thus, the placenta is not only central to fetal development but also key to understanding and preventing fetal demise.
